# Potential Pitfalls With Automatic Sentiment Analysis: The Example of Queerphobic Bias

**DOI:** 10.1177/08944393231152946

**Published:** 2023-02-02

**Authors:** Eddie L. Ungless, Björn Ross, Vaishak Belle

**Affiliations:** 1 Ringgold: 151022 The University of Edinburgh, Scotland, UK

**Keywords:** sentiment analysis, AI bias, natural language processing, queerphobia

## Abstract

Automated sentiment analysis can help efficiently detect trends in patients’ moods, consumer preferences, political attitudes and more. Unfortunately, like many natural language processing techniques, sentiment analysis can show bias against marginalised groups. We illustrate this point by showing how six popular sentiment analysis tools respond to sentences about queer identities, expanding on existing work on gender, ethnicity and disability. We find evidence of bias against several marginalised queer identities, including in the two models from Google and Amazon that seem to have been subject to superficial debiasing. We conclude with guidance on selecting a sentiment analysis tool to minimise the risk of model bias skewing results.

Natural Language Processing (NLP) tools are being increasingly adopted in the social sciences (Robila & Robila, 2020; Saifee et al., 2020), the hope being that cutting-edge technologies will allow researchers to analyse data with efficiency and a human-like understanding of language use. However, social biases embedded in these models threaten to undermine this hope (Blodgett et al., 2020; Shah et al., 2020). For example, sentiment analysis tools developed using deep learning techniques have been shown to reflect biases such as racism, sexism (Kiritchenko & Mohammad, 2018) and ablism (Hutchinson et al., 2020), through use of a template-based approach which we likewise adopt in this paper. We expand on existing work by testing for queerphobia^1^ in six popular sentiment analysis tools. Sentiment analysis is used to assess the prevailing tone in political discussions online (Hagen et al., 2022); for mental health triaging (Aladağ et al., 2018); to quantify product success (Li et al., 2018); to compare human and bot tweets (Antenore et al., 2022). If these models systematically give different scores depending on the presence of queer identity terms, this threatens to undermine research conducted using these tools.

Our carefully constructed data set of 29,472 sentences (examples given in Table 1) allows for precise comparison across queer identities: a template structure is used to minimise the impact of confounding linguistic variables, which allows us to pinpoint which terms the models show bias against. As Smith et al. (2022) write, ‘for social applications of NLP, it’s crucial to know what’s in your data ... handcrafting data … affords more control’. We take our approach (and templates) from Kiritchenko and Mohammad (2018), who were able to demonstrate bias against women and Black people in the majority of the 200 deep learning-based sentiment analysis tools they tested, by permuting which names appeared in the templates.

**Table 1.** Table of scores for three example sentences. Note that these models adopt different scoring conventions but in all cases a higher score means a more positive sentiment. All models show a variation in scores depending on the identity phrase.

Template		Model					
		Google	Amazon	IBM	LIWC	VADER	AFINN
<identity phrase > feels enraged.		––					
Variant	This person feels enraged.	−0.20	0.04	−0.36	74.76	−0.40	−2
	This latinx* person feels enraged.	−0.10	0.04	0.25	43.37	−0.40	−2
	This same gender loving* person feels enraged.	−0.40	0.55	−0.36	13.15	0.30	0

*terms defined in Methods.

In addition to three proprietary deep learning models – at the cutting edge of sentiment analysis – we also test three lexicon-based approaches, which rely on hand-engineered wordlists labelled for valence. Valence decisions can be subjective, meaning bias is likely to impact labels (Mohammad, 2017), but these approaches offer far greater transparency, so the source of bias can be identified (and counteracted) with relative ease. Illustrating this point, in the SentiStrength (Thelwall et al., 2010) lexicon both ‘gay’ and ‘queer’ have negative sentiment labels. This contrasts with the ‘black box’ nature of modern deep learning approaches where carefully constructed probes are needed to ‘tease out’ patterns of bias (Bender et al., 2021), and addressing this bias involves more complex processes (Meade et al., 2021). The six tools we test in this paper sit at either of end of the spectrum from highly interpretable to fully ‘black box’ systems.

Our findings are intended to provide guidance to those selecting a tool for research or commercial purposes. We focus on sentiment analysis as this has a wide range of applications, but our paper can be thought of as a cautionary tale for adopting NLP technologies in research. Our focus on queerphobia allows us to demonstrate the importance of a nuanced approach to identity, as many queer identities exist at the intersection of multiple forms for marginalisation, and a binary comparison between queer and non-queer identities (parallel to comparing white and Black names) is not sufficient for understanding how the power dynamics associated with sexuality, gender and ethnicity might interact and impact how certain terms are treated by these tools.

In the following, we discuss some of the existing uses of sentiment analysis tools and the role deep learning approaches will likely play in future. We discuss how sentiment analysis tools might come to be biased and how this reflects larger problems for those who use NLP tools in their research. We then present and test the six selected tools and discuss our findings, and their implications for researchers and commercial users of sentiment analysis tools and other NLP technologies.

## Background

### Sentiment Analysis Approaches

Current approaches to sentiment analysis can be roughly categorised into lexicon-based approaches and machine-learning (ML)-based approaches. Lexicon-based approaches use dictionaries of words and phrases labelled for sentiment, and typically include rules to deal with negation or use of modifiers. They use scoring mechanisms to determine the sentiment of a span of text, for example, taking an average. Lexicon-based approaches are efficient to implement as they do not require training data, are easy to use and produce results almost instantly, making them ideal tools for research. Lexicon-based approaches are usually very transparent – it is easy to understand how the sentiment score was determined, based on the relevant words in the text.

ML-based approaches involve training a system to automatically classify or score spans of text for sentiment. They require training data, which is often manually labelled for sentiment, making them resource intensive. Many different techniques fall under the umbrella of machine learning. A nearest neighbour classifier would look to the training instances most like the test instance to determine its sentiment. One type of ML-based approaches are deep learning models, which are trained on vast amounts of training data to make labelling decisions based on abstract features. Examples of proprietary deep learning systems for sentiment analysis include IBM Watson’s Natural Language Understanding service.^2^ Such models can be deployed without additional model training, so do not require a background in computer science.

### Literature Review

A search of the SCOPUS^3^ and ASSIA^4^ databases for ‘sentiment analysis’ shows that automated tools are widely used in the social sciences to conduct research on topics as diverse as understanding the impact of COVID and other disasters (Kaur et al., 2020; Razavi & Rahbari, 2020); to analysing the online behaviours of vulnerable groups (Saifee et al., 2020). Much recent work relies on lexicon-based approaches such as LIWC (Tausczik & Pennebaker, 2010), VADER (Hutto & Gilbert, 2014) and SentiStrength (Thelwall et al., 2010), likely due to their ease of deployment. Clearly, simple lexicon-based approaches still play a significant role in social science research.

However, lexicon-based approaches face several major problems. They risk becoming outdated if they fail to include recently coined terms, which are particularly prevalent in casual online discourse (Hilte et al., 2018). As stated above, sentiment labels are largely subjective (Mohammad, 2017). Related to these two points, marginalised groups may be impacted if the lexicon does not include terms from non-standard English(es) (such as African American Vernacular English) or if these terms are included but labelled by individuals who do not belong to the community. This will be starkly evident in the case of (the many) identity terms that are reclaimed slurs, as with ‘queer’ being rated as negative in SentiStrength. Content produced by queer people may be inaccurate as a result. Finally, lexicon-based approaches are overly simplistic – they will often fail to properly consider negation, modals and sentence structure, as well as sarcasm and humour (Mohammad, 2017).

These problems hamper the performance of lexicon-based approaches, and they are increasingly being abandoned in favour of ML models which outperform on benchmark sentiment analysis datasets (Poria et al., 2020). It has been predicted that use of ML will become the norm in social science research (Robila & Robila, 2020). Examples of papers already using ML approaches include Kaur et al. (2020) and Li et al. (2020). Deep learning models can offer more sophisticated analysis having learned abstract patterns from vast amounts of data. However, this greater accuracy comes at the cost of less transparency. The values of deep learning models’ parameters are rarely interpretable, meaning it is unclear how classification decisions are made.

A further problem with ML-based tools is that they often pick up on ‘spurious associations’ (Utama et al., 2020) between group identifiers and negative characteristics, an artefact of training data which reflects human biases. This means these systems may develop a predictive bias against marginalised groups (Shah et al., 2020), consistently producing a different output due to the presence of identity terms or community-specific language use. The interested reader should refer to Shah et al. (2020) for an overview of the four main ways that NLP models develop predictive biases. There is substantial evidence of predictive bias in NLP across a range of tasks, for example, hate speech detection (Röttger et al., 2020) and coreference resolution (Cao & Daumé, 2020; Rudinger et al., 2018), in addition to sentiment analysis (Hutchinson et al., 2020; Kiritchenko & Mohammad, 2018). Attempts have been made to combat such bias, but despite this many commercially available tools show evidence of bias, including against queer individuals (Buolamwini & Gebru, 2018; Röttger et al., 2020; Thompson, 2017). This leads us to predict that deep learning-based sentiment analysis tools will show a predictive bias against queer terms.

Such predictive bias can lead to harm being done to marginalised groups. Barocas et al. (2017) divide the potential harms of NLP systems into representational and allocational harms. The former refers to harms done through the misrepresentation of a group, including through differences in system performance. A group may be misrepresented as having polarised views about a topic because the terms they used to talk about their lives result in inaccurate scores (in the sense that the presence of terms such as ‘same gender loving’ can lead to a difference in score despite the intended sentiment being the same, as shown in Table 1 for IBM). ‘Allocational harms’ refers to the unfair allocation of resources to the demographic, for example, access to job opportunities, funding etc. Use of a biased sentiment analysis tool may lead to allocational harms if sentiment analysis is used as a form of triaging, for example, to determine the success of an initial mental health intervention and thus select participants for further treatment. Resources may be unfairly allocated if biased sentiment analysis tools give inaccurate scores to certain communities.

Both lexicon- and deep learning-based approaches have the potential to cause harm to marginalised groups due to inaccurate scores, for the reasons outlined above, and so we investigate both kinds of tools as to whether they show bias against marginalised queer identities. The NLP literature suggests we will find evidence of a predictive bias against marginalised queer identities in the deep learning models, whereby they will receive lower sentiment scores (as women and Black people receive lower scores in Kiritchenko & Mohammad (2018)). The presence of ‘gay’ and ‘queer’ in SentiStrength suggests other lexicon-based approaches may similarly show a negative bias against queer identities.

Higher scores in one group may indicate their ratings have been inflated by the presence of certain identity terms, or that the scores for the other group have been reduced for the same reason. Either way, the system is erroneously basing sentiment rating on the presence of identity terms not intended to indicate sentiment. It could be said the models are biased against the less marginalised identity groups if these also receive inaccurate scores. However, we formulate our hypotheses around bias against the marginalised groups because they are historically disadvantaged. Further, we test monodirectional hypotheses, predicting that more marginalised groups will receive more negative sentiment ratings, but it is important to note that inaccuracies in either ‘direction’ can cause harms, for example, if such models are used in the context of mental health triaging.

Our primary hypothesis (H1) is that sentiment analysis tools will show an overall bias against queer identities compared to non-queer identities, in that queer identities will receive more negative sentiment ratings. Inspired by an intersectional approach to identity (Crenshaw, 1989), we hope to provide a more thorough evaluation of the tools by also looking at marginalised groups within the queer community: treating queer identities as a homogeneous group could obscure bias against particular queer identities such as those specific to people of colour. We predict that these systems will reflect bias against marginalised groups within the queer community (H2), for example, there will be greater bias against queer women compared to other queer identities, because women additionally experience sexism. To explore H2 we compare female and male (H2A), transgender and cisgender (H2B), and ethnicity-specific and non-specific identities (H2C) within the queer community, predicting that in each case the former group will receive more negative sentiment ratings than the latter, because they are further marginalised by their gender, trans status and ethnicity, respectively.

## Method

### Dataset Creation

We used 10 of the templates, and the emotional vocabulary from Kiritchenko & Mohammad (2018). We modified the templates to include a combination of up to three identity terms about trans status, sexuality and gender (given in Table 3) in that order. Examples of the templates, identity phrase combinations and emotional terms (and the resulting sentences) are given in Table 2. Use of templates allows for careful comparison across different identities. Using natural data would have introduced many confounding variables including use of slang and other dialectal differences across communities. We would likely struggle to find truly comparable data between mainstream queer identity terms such ‘gay’, identity terms used by people of colour such as ‘same gender loving’ and identity terms that have not entered the mainstream such as ‘demisexual’. For the purposes of careful comparison across intersecting identities, a template-based approach provided the best option.

**Table 2.** Table showing how templates, identity phrase combinations and emotional terms were combined to create the sentences in our data set.

Template	Identity term combination	Emotional term	Sentence
I saw this <identity phrase> in the market.	bisexual two-spirit person	N/A	I saw this bisexual two-spirit person in the market.
This <identity phrase> told us all about recent <emotion> events.	lesbian woman	wonderful	This lesbian woman told us all about recent wonderful events.

To establish our list of identities, we started with relevant terms from Dixon et al. (2018) (indicated in Table 3 in bold). We expanded on this list to represent more diverse queer identities, for example, including three additional terms specific to people of colour. Below, we focus on those terms relevant to our hypotheses. Further details on all the identity terms we chose can be found in the Appendix.

**Table 3.** Table showing identity terms including in our data set.

Trans status	Sexuality	Sexuality presentation	Gender
Cisgender	Asexual	Bear (m)	**Woman (f)**
**Transgender**	**Bisexual**	Butch (f)	**Latinx**
	Demisexual	Cub (m)	**Man (m)**
	Fluid	Dyke (f)	**Non-binary**
	**Gay**	Femme (f)	Transfeminine
	**Heterosexual**	Stud (f)	Transmasculine
	**Homosexual**	Twink (m)	Two-spirit
	**Lesbian (f)**		
	**LGBT** ^*i*^		
	**LGBTQ** ^*i*^		
	Pansexual		
	**Queer** ^*i*^		
	Same gender loving		
	**Straight**		

Note. Bold font indicates identity terms from Dixon et al., 2018. Original list includes ‘nonbinary’ where we use the more popular spelling ‘non-binary’. Original list includes ‘female, male’ rather than ‘woman, man’.

Underline indicates the term is ethnicity specific.

(m) indicates this identity is typically used by men. (f) indicates this identity is typically used by women.

I understood as an umbrella term for non-heterosexual sexualities

Note that if unspecified, the norm is assumed, because marked identities use marked language (Bucholtz & Hall, 2004; DePalma & Atkinson, 2006). Non-normative identities such as non-heterosexual, non-cisgender or asexual identities typically must be explicitly stated to be understood. If these identity terms are not stated, then it is assumed the norm applies: when sexuality is not given, heterosexuality is assumed; when trans status is not given, cisgender identity is assumed. However, the data set includes explicitly normative identity terms such ‘straight, cisgender’, which allows for comparison between queer identities and both the explicit and assumed norms.

Our data set is designed to evaluate how sentiment analysis tools treat sentences about individuals belonging to different marginalised groups within the queer community. For example, queer women will face additional discrimination to queer men, even experiencing misogyny from within the queer community (Hale and Tomas, 2018): it is likely such bias will be evidenced in the models, with sentences about queer women receiving lower scores than those about queer men. To enable a thorough exploration of the impact of queer female identities on sentiment ratings, we include ‘butch, femme, dyke’ and ‘stud’, in addition to ‘lesbian’ from Dixon et al. (2018). For queer men, we include the related terms ‘bear, cub, twink’. These terms all relate to presentation-driven subgroups within the non-heterosexual community.

Transgender individuals face related gender oppression, from both outside and within the queer community (Iantaffi & Bockting, 2011; Stone, 2009). To identify predictive bias against transgender individuals (our hypothesis H2B), we include identities with different trans status (‘transgender, cisgender’, or trans status is not given and cisgender is assumed). We also include a number of non-binary identity terms, under the umbrella of transgender identities,^5^ namely ‘transfeminine, transmasculine, two-spirit’ in addition to ‘latinx, non-binary’ from Dixon et al. (2018). The scarce research comparing binary and non-binary transgender people's experiences suggests they face similar levels of victimisation (Rimes et al., 2019).

Many queer identity terms are not adopted by people of colour, due to feelings of alienation (Battle, 2002). Individuals may adopt alternative terms; we include a small number of these in our data set to test for bias against queer people of colour. The terms we include are ‘stud’ (a term used by some black women who love women with a particular masculine aesthetic (Lane-Steele, 2011)); ‘same gender loving’ (a term used by Black individuals attracted to those of the same gender, possibly in addition to those of another gender (Battle, 2002)); and ‘two-spirit’, an umbrella term for third gender identities unique to Native Americans (Anhorn, 2016),^6^ in addition to ‘latinx’ from Dixon et al. (2018) (a term used by non-binary individuals of Latin American descent (Cristobal Salinas, 2020)). We include these identity terms when comparing ethnicity-specific to non-ethnicity specific queer identities (H2C).

The key challenge in creating this data set was combining identity terms in an appropriate manner: the identity terms could not all be combined (e.g. ‘cisgender’ and ‘transmasculine’). Sensitively combining identity terms required extensive research. Researchers wishing to explore additional combinations can do so using the source code provided.

Some of the combinations of terms, for example, ‘transgender same gender loving woman’ may be unlikely to occur together in natural data, an artefact of using templates. However, all are valid identities. It is our belief that how common an identity is written about should not determine whether we test for bias against that identity, the implicit decision made by researchers testing only the most salient identities. By testing many combinations, we are able to determine if some of the combinations of terms interact in unexpected ways, for example, resulting in much higher or much lower scores than would be expected given how the system treats the terms individually.

The 30 identity terms were combined with the templates (along with ‘this’ plus ‘person’, ‘woman’ or ‘man’ where appropriate) to give 29,472 sentences. We designed our data set such that many forms of predictive bias can be identified. In the present paper, we focus on four likely examples of predictive bias, but our data set allows for the exploration of biases against many marginalised queer identities. Of course, our list of identities is not comprehensive; the program that generates the data set can handle additional terms in an appropriate manner.

### Selecting Sentiment Analysis Tools

We focus on popular sentiment analysis tools that are likely to be used by researchers beyond computer science: three that use deep learning and three with a lexicon-based approach. The three deep learning products we consider are from Google, Amazon and IBM, all of which offer ‘out-of-the-box’, pre-trained sentiment analysis tools, namely Google Cloud Natural Language, Amazon Comprehend and IBM Cloud Natural Language Understanding. These are three of the world’s biggest cloud service providers, meaning their products are widely available.

To establish our list of lexicon-based approaches, we looked at the proprietary tools identified in our survey of the ASSIA database (described in the Literature Review). The tools were VADER, LIWC, NRC (Mohammad & Turney, 2013), SentiStrength, AFINN (Nielsen, 2011), Textblob (Loria, 2018) and Semantria.^7^ VADER and LIWC were the first and second most common tools, so we examine them both in this paper. NRC and AFINN were used by two papers. Ultimately, we opted to investigate AFINN because it is older, although still used in recent papers such as Borakati, (2021), and so we felt it was more likely to record queer terms as negative and be potentially biasing contemporary findings. We also found VADER, LIWC and AFINN to be widely used by papers in the SCOPUS database.

We test only a subset of the sentiment analysis tools in popular use. We make our data set publicly available for use in testing other models (see Supplemental Material).

### Testing Procedure

To identify the impact of identity terms we compare between different variations (with different identity term combinations) of a particular template plus emotional term (henceforth, template(s)^e^). For example, we compared the sentiment rating for when the template^e^ ‘This < identity phrase > told us all about recent wonderful events’ was combined with ‘lesbian woman’, ‘bisexual two-spirit person’, ‘person’ and other identity term combinations. This allowed us to identify the impact of the identity terms on the ratings given by the tools.

To facilitate the testing of our hypotheses, we grouped multiple identities together where appropriate and considered the average rating across all templates^e^ for identity term combinations in this grouping. This allowed us to effectively identify broad patterns in how identities were treated by the systems. For example, to test for bias against queer identities (H1), we grouped all the identity combinations that included any terms other than ‘cisgender, heterosexual, straight’ into a queer group, and grouped the remaining identity combinations in a non-queer group. We then conducted a two-tailed paired sample t-test between the average rating for queer versus non-queer identities across all the templates^e^. We followed this same procedure for all queer female and queer male identity combinations (H2A) – that is, we compared all identity combinations that included a queer female identity term such as ‘lesbian’, or a queer identity term such as ‘bisexual’ plus ‘woman’, to all identity combinations that included a queer male identity such as ‘twink’ or a queer identity term plus ‘man’. We did the same for all transgender and all queer cisgender identity combinations (H2B). For queer ethnicity-specific identity combinations we compared them to the non-ethnicity-specific identity terms closest in meaning (H2C).

Where appropriate, we conduct additional analyses, for example, looking at minimally contrasting pairs such as ‘I saw this bisexual man’ versus ‘I saw this bisexual woman’. For the lexicon-based approaches we also looked at the lexicon itself, where it was available, or at the scores assigned to individual words.

## Results

In this section, we look at the results of testing each of the six tools with our novel dataset. Though only LIWC showed a systematic bias against queer identities, giving them consistently lower sentiment ratings, marginalised groups within the queer community might still be impacted by inaccurate results from all of the tools we tested. We give a summary of our results in Table 4.

**Table 4.** Table demonstrating which hypotheses are supported by our analysis of each model. For H2B, ‘partial support’ indicates systematic bias against some transgender identities, that is, only binary or only non-binary transgender.

	Model					
Hypothesis	Google	Amazon Comprehend	IBM	LIWC	VADER	AFINN
H1				Supported		
H2A	Supported	Supported				
H2B		Partial Support	Partial Support	Partial Support		
H2C						

### Google

Google assigned sentiment ratings between –1 and 1, where –1 indicates a very negative sentiment and +1 indicates a very positive sentiment. All our hypotheses predict that the more marginalised group will receive a more negative sentiment rating; for Google, this will be indicated by a lower sentiment score.

Following our paired t-test, we found queer identities were given a significantly higher rating than non-queer identities: –0.218 compared to –0.246, t(123) = 3.68, *p* < .001. The was counter to our hypothesis **H1**. However, there was some evidence of bias against groups within the queer community, in line with H2, which we explore below.

We found female queer identities were rated lower than male (**H2A**): –0.250 compared to –0.201, t(123) = -4.72, *p* < .001. Further, where like-for-like comparisons are possible, such as ‘bisexual man’ versus ‘bisexual woman’, the latter received a lower score on average. This suggests a bias against queer women compared to queer men.

Regarding transgender identities, we found neither ‘transgender’ nor ‘cisgender’ had any impact on sentiment score (sentences with these terms received identical scores to those with no trans status terms), meaning binary transgender identities did not receive more negative sentiment ratings than queer cisgender identities. Further, we found non-binary identities were rated as less negative than queer cisgender identities: –0.212 compared to –0.230, t(123) = 2.99, *p* < .005. Thus, we found no support for hypothesis **H2B**.

Comparison between the terms used by people of colour and those most closely comparable non-ethnicity-specific terms gave no support for **H2C**: the terms used by people of colour elicited similar or more positive ratings. For example, same gender loving woman received a more positive sentiment rating than lesbian woman.

The results for Google suggest a level of superficial debiasing may have been conducted. For some of the terms, there were no differences in score whether a sentence contained the term or not. This suggests the system may have been ‘instructed’ to ignore certain identity terms. In Table 5, we give results across 13 sexualities plus the term ‘man’ to illustrate our point. Google Cloud is effectively oblivious to terms including ‘straight, gay, queer, asexual’. Table 5 illustrates the pattern that less common terms seem unlikely to be included in a purported ‘ignore list’: smaller minorities within the queer community, often the most marginalised, are still impacted by spurious differences in score. Another major issue with use of an ignore list to avoid bias is that some identity terms are crucial to contextualising other words used in the sentence. Within a (particularly Black and Latinx) queer context, the phrase ‘sickening’ means something very positive (Calder, 2019). In non-queer contexts, this term is typically very negative in sentiment. Use of an ignore list means the system will be oblivious to these contextual clues.

**Table 5.** Table showing mean sentiment rating across select male identities, for the three deep learning-based sentiment analysis tool, alongside the frequency of the terms in two data bases to demonstrate that popular terms are more likely to be standardised.

Identity + man	Google mean	Amazon mean	IBM mean	Wikipedia N-gram count	Google N-gram %
	−0.225*	0.613*	−0.340	351,972	6.23e-2
Straight	−0.225*	0.613*	−0.354	630	9.62e-6
Gay	−0.225*	0.613*	−0.540	282	3.31e-5
Homosexual	−0.225*	0.613*	−0.401	132	1.83e-6
Heterosexual	−0.225*	0.613*	−0.624	53	2.55e-6
Bisexual	−0.225*	0.613*	−0.524	47	9.67e-7
Queer	−0.225*	0.613*	−0.448	5	2.52e-6
Asexual	−0.225*	0.590	−0.374	3	4.24e-8
Pansexual	−0.225*	0.613*	−0.505	1	0
LGBT	−0.225*	0.613*	−0.350	1	0
LGBTQ	−0.225*	0.613*	−0.355	0	0
Demisexual	−0.240	−0.349	−0.349	0	0
Fluid	−0.090	−0.166	−0.166	1	5.21e-8
Same gender loving	−0.086	−0.357	−0.357	Unavailable	0

Here we report only the negative confidence score for Amazon.

*No difference in variation between other scores marked with * in column, standard deviation of error = 0.

### Amazon

Amazon gives two separate confidence ratings, between 0 and 1, for the sentence being of positive or negative sentiment. A more negative sentiment rating would be indicated by a) a lower confidence rating for positive sentiment and b) a higher confidence rating for negative sentiment.

We found queer identities were rated as slightly less likely to be positive, but this was not significant (**H1**). Queer sentences were rated as significantly less likely to be negative, 0.552 compared to 0.614, t(123) = –7.15, *p* < .001. Thus, we found no support for H1. However, as with Google we did find evidence of bias against marginalised groups within the queer community, in line with H2.

Female queer identities were rated as more likely to be negative and less likely to be positive than male (**H2A**). For positive confidence rating, female queer identities received 0.276 compared to 0.287, t(123) = –4.14, *p* < .001. For the negative confidence rating, 0.590 compared to 0.580, t(123) = 5.45, *p* < .001. Thus, we found support for hypothesis H2A.

As with Google we found neither ‘transgender’ nor ‘cisgender’ had any impact on score (**H2B**). Non-binary gender identities were rated as significantly less likely to be negative, 0.573 compared to 0.579, t(123) = –4.64, *p* < .001. However, they were *also* rated as significantly less likely to be positive than cisgender identities, 0.277 compared to 0.280, t(123) = –3.18, *p* < .005. Thus, we found some limited support for H2B.

None of the terms used by people of colour received lower positive confidence ratings or higher negative confidence ratings than their closest non-ethnicity-specific equivalents (**H2C**). For example, ‘stud’ was rated as more likely to be positive and less likely to be negative than ‘butch’. ‘Two-spirit, non-binary’ and ‘latinx’ received totally identical ratings, again suggesting the existence of an ‘ignore’ list, though this was not the case for the Google results.

As with Google, Amazon’s system seemed to have been subject to the same kind of superficial debiasing – see Table 5. The two systems do not ignore the same terms, as evidenced by the scores assigned to ‘asexual’, ‘fluid’ (see Table 5) and ‘two-spirit, non-binary’ and ‘latinx’ (see above).

### IBM

As with Google, IBM assigns sentiment ratings between –1 and 1, where a lower score indicates a more negative sentiment.

For IBM, we found queer identities were rated significantly higher than non-queer identities, –0.409 compared to –0.444, t(123) = 4.17, *p* < .001. Therefore, we did not find support for **H1**.

Female queer identities received a slightly more negative rating than male queer identities on average (**H2A**), though we found this was not significant, perhaps indicative of successful debiasing, although it may be the training data IBM uses means their model is less prone to gender bias.

Binary transgender identities were rated as less positive than explicitly cisgender identities (those identity combinations where ‘cisgender’ is included) and assumed cisgender identities, where trans status is not mentioned and the norm is assumed (**H2B**). Binary transgender identities were rated –0.510 compared to –0.445 for explicitly cisgender identities, t(123) = –7.01, *p* < .001. Binary transgender identities were rated –0.510 compared to –0.422 for assumed cisgender identities, t(123) = –9.26, *p* < .001. In comparison, non-binary identities were rated more positively (–0.401) than queer cisgender identities: higher than explicitly cisgender identities, t(123) = 5.93, *p* < .001; and higher than assumed cisgender identities, t(123) = 2.92, *p* < .005.

There was some evidence of bias against queer people of colour (**H2C**). For example, ‘latinx’ was rated significantly more negatively than ‘non-binary’, –0.416 compared to –0.393, t(123) = –2.85, *p* < .005 . Similarly, ‘same gender loving’ was rated as significantly more negative (–0.462) than ‘homosexual’ –.406, t(123) = –3.95, *p* < .001, though it was also rated as significantly more positive than ‘gay’, -0.502, t(123) = 3.4, *p* < .005. However, there was no overarching pattern of bias against ethnicity-specific terms.

IBM had not been subject to the same proposed heuristic debiasing as the other two deep learning models, and this is evident in the more varied results compared to Google and Amazon in Table 5.

### LIWC

LIWC assigns an emotional tone score between 0 and 100, where less than 50 indicates a negative sentiment. Because LIWC explicitly labels which terms are positive and which are negative, we supplemented statistical analysis with qualitative exploration of the dictionary and of the tone scoring system. Of all the identity terms, only ‘loving’ (from ‘same gender loving’) was included in the LIWC dictionary as explicitly labelled for positive emotional tone; none were labelled for negative emotion. We did find others did differ in other regards, such as whether they were marked as ‘informal’, which may have influenced their overall LIWC emotional tone rating.

Queer sentences were rated as less positive than non-queer sentences (**H1**), largely due to sentence length: a linear regression analysis found that sentence length accounted for almost a quarter of overall variation (*R*^2^ = .225). Marked identities use marked language and usually must be explicitly ‘spelled out’ to be understood, meaning sentences about queer identities will often be longer. This seems to be connected to the fact that LIWC records the proportion of a sentence that a particular word category makes up (LIWC categorises words according to their inclusion in word lists such as *articles* but also *positive emotion words* (Tausczik & Pennebaker, 2010). Sentences including multiple identity terms might then have proportionally fewer positive terms resulting in lower scores, even if the identity terms themselves have no valence associated with them.

In addition to this general pattern of bias against queer identities largely because of sentence length, we found evidence of bias against marginalised groups within the queer community (**H2**). Neither ‘woman, man’ nor ‘person’, nor any of the sexuality presentation terms had any impact on score, meaning there was no bias against queer women. Implicitly cisgender identities received higher scores likely due to the impact of (shorter) sentence length. Same gender loving received more negative scores on average than ‘lesbian’, ‘gay’ or ‘homosexual’, likely an artefact of length, despite the fact loving is explicitly tagged as having positive emotion. However, there was no overall pattern of bias against ethnicity-specific terms.

In conclusion, whilst none of the identity terms were labelled as positive or negative, by virtue of marked identities using marked language, some marginalised identities were subject to bias by LIWC, giving support for H1 and partial support for H2B.

### VADER and AFINN

VADER outputs three scores between 0 and 1, which sum to 1, indicating how likely a sentence is to be positive, negative or neutral (based on the sentiment of the words in the sentence), and a single compound score between –1 and 1 indicating the overall sentiment. AFINN is one of the most simple sentiment tools; it assigns a score between –5 and 5, the average sentiment of words in the sentence. As with LIWC, VADER and AFINN explicitly label which terms are positive and which are negative, so we tested our hypotheses through an exploration of the dictionaries and of the sentiment scoring systems. Whilst these simple models benefit from being highly transparent, they are also unable to deal with more complex sentence structure, meaning their scores may be very inaccurate for reasons other than the presence of queer identity terms.

For both VADER and AFINN’s emotional lexicons, only ‘straight’, ‘loving’ (as in ‘same gender loving’) and ‘spirit’ (as in ‘two-spirit’) are present, all recorded as positive. Sentences containing these words received slightly more positive scores. However, there was no overall pattern of bias against queer identities (**H1**) or even marginalised groups within the queer community (**H2**), though these identities may be subject to artificially positive sentiment scores. Unlike for LIWC, sentence length had no impact for AFINN or VADER, meaning there was no systematic bias against marked, non-normative identities.

## Discussion and Limitations

Our results indicate that all six tools we tested had the potential to give inaccurate results based on the language people use to talk about their lives. Only one model, LIWC, showed an overall negative predictive bias against queer identities compared to non-queer, a result of ‘penalising’ marked identities. However, LIWC, Google, Amazon and IBM all showed a negative bias against marginalised groups within the queer community. For example, queer women received a lower score on average compared to queer men from Amazon and Google. Whilst we do not give specific recommendations for the best tool to select (as this depends on a variety of factors including a researcher’s technical abilities, and there are many more tools available than those we selected to test), in the following we provide some guidance for choosing an appropriate sentiment analysis tool for the task and what steps can be taken to mitigate the impact of predictive bias.

Counter to our predictions, more marginalised groups did not always receive lower scores – in some cases the marginalised identities received much higher scores. Although this does not support our hypotheses, inaccurate scores in either ‘direction’ can be harmful; individuals may be unfairly excluded from opportunities or misrepresented in research because they receive systematically higher or lower scores. If reference to a person’s non-binary gender identity results in systematically higher sentiment ratings (as is the case for Google), these individuals may be considered lower priority in a mental health triaging system, for example. Use of a sentiment analysis tool should be complemented with further analysis, as a sanity check, for example, of how different demographics are distributed across rating brackets, to identify patterns of systematic differences, some of which may be spurious.

For the lexicon-based approaches we tested we were largely able to pinpoint the sources of bias. Where identity terms were included in the lexicons, we were able to easily identify this. In this case, a solution might be to remove words, or introduce rules such as that ‘loving’ in the context of ‘same gender loving’ should not be considered positive. This would require sensitive revisions to the existing lexicons and associated rule systems. Constructing a lexicon is very resource intensive and ensuring that it is culturally sensitive adds to this workload. However, without this work many researchers currently using these lexicon-based approaches may receive inaccurate results due to certain identity terms occurring in the lexicon.

For VADER and AFINN, the scale of the issue seems relatively limited in that only a handful of identity terms are included in the lexicon. However, we encourage researchers to spend some time checking that terms relevant to their participants’ lives do not feature in unexpected ways in the lexicon they are using, and where possible even make edits to the lexicon to remediate this; this of course applies to other identities beyond queer ones. This is particularly relevant when data are gathered from a diverse range of participants, where some will be using the terms and others will not be, or when use of reclaimed slurs is common. For a fair and insightful comparison, the impact of identity terms must be removed. The major benefit of lexicon-based approaches is their transparency, which makes this kind of investigation and remediation easy. This comes at the expense of the system being able to process complex sentence structure, as these tools are far less sophisticated than the deep learning approaches. However, this sacrifice may be necessary in order to be confident that marginalised identity terms are not impacting the sentiment score. It is hard to have this same confidence in deep learning based approaches without the use of carefully constructed probes (Bender et al., 2021) which only exist for a small number of identities (Blodgett et al., 2020).

For LIWC, the issue of predictive bias seems to be more pervasive. We found that because LIWC factored in sentence length, some marginalised identities would be subject to predictive bias. The seemingly innocuous decision to incorporate sentence length into sentiment calculations means LIWC shows bias against marked identities. If a researcher is comparing queer and non-queer individuals, LIWC could introduce a predictive bias against queer individuals if they frequently use marked identity terms when they write, and the non-queer participants do not. This same caution would apply to any linguistically marked (non-normative, marginalised) identities, for example, people of colour or people with disabilities.

For the deep learning approaches, our use of templates allows for careful comparison across terms, and we were able to identify that certain identity terms have a negative impact on score, for example, many of the terms associated with female queer identities (in the case of Google and Amazon). Some of the marginalised identity terms also resulted in significantly more positive ratings, for example, ‘same gender loving’ resulted in some of most positive ratings from Amazon; despite offering more sophisticated language processing abilities, just like the lexicon-based approaches Amazon (and Google) gave inflated scores to ‘same gender loving’ identities, likely because of the presence of the word ‘loving’. Whilst these models are designed to consider context, they appear to be failing to do so here, likely due to a lack of diverse training data. Further, the fact that scores are likely to vary most for the least common identities (which we took as evidence of heuristic debiasing) suggests that these tools are to be avoided when considering data from or about individuals belonging to highly marginalised communities.

The heuristic debiasing approach that we suspect Google and Amazon have adopted does mean more mainstream queer and non-queer identities receive comparable scores, but in trying to rid the model of predictive bias against marginalised identities, the developers have also inhibited the models’ ability to use ‘semantic bias’ to contextualise the meaning of slang terms. In addition to the ‘sickening’ example given in Results, there are countless examples of words that have different sentiments across different queer communities, including ‘fierce’ (Calder, 2019) (a positive sentiment in drag/ballroom culture, typically a negative sentiment outside of this).

Further, the lack of transparency or of easy modification is an issue for deep learning approaches. Due to their black box nature, it is not clear why some identities were rated higher than others by these models (and the fact they are proprietary models further limits investigation), though we detail possible sources of predictive bias in the Literature Review. It is likely that significant differences in the distribution of terms in the training data resulted in these differences, as the systems learned to ‘focus’ on these terms as an almost ‘heuristic’ way of assigning score (Zhao et al., 2017). There is a push for better documentation of potential biases in models (Mitchell et al., 2019), but often bias is detected (by the impacted individuals (Buolamwini & Gebru, 2018)) after the models are deployed. This leaves social scientists wishing to use these models to collate various analyses and sift through model documentation that may not explicitly address the issues of bias, in order to understand the likely impact of bias in their own results. As mentioned above, model bias will be a particular issue when considering data from participants belonging to different communities whereby use of identity terms by one group will systematically alter their results; unlike with lexicon-based approaches, it is not always easy to identify which terms will lead to bias without testing with a data set such as ours. There is a significant body of work looking to develop less biased language models for a range of tasks (Dixon et al., 2018; Liang et al., 2020; Schick et al., 2021; Ungless et al., 2022; Webster et al., 2021; Zhao et al., 2018), for example, using counterfactually augmented data (Sen et al., 2021), which researchers with the right technical skills may be able to adopt, where they have access to the original model. However, for those who must rely on third party tools, our findings suggest marginalised individuals continue to be impacted by predictive bias despite the likely use of debiasing strategies, and in particular the least salient identities.

This paper faces its own such limitations: we use a limited set of identities to try to measure bias against a potentially limitless community (in the sense that new identity terms are constantly being adopted). By including a relatively diverse set of queer identities, we can indicate some of the issues individuals might face due to use of automated sentiment analysis tools. However, we can only hope to approximate bias against queer people. Future work is needed to expand on the coverage to more identities. A potentially fruitful line of research looks to identify bias without relying on a predefined list of identity terms (Utama et al., 2020) which could avoid the issue of only salient identities being investigated.

In summary, our results suggest a three-step strategy that users of sentiment analysis tools – in both industry and academia – should follow.

1. **Bias audit:** Before selecting a tool, they should proactively audit the tool for potential bias. This includes the identification of groups that might be negatively affected by inaccurate results. For example, in film reviews, it would be problematic to misclassify a review as negative when queer people are the subject of the film. The sentiment analysis model or tool should then be scanned for bias against these groups with the help of a dataset designed for this purpose. For queer identities, our dataset can be used; for other identities, such datasets will yet have to be created.
2. **Sanity check**: Then, after using the selected tool to calculate sentiment on a dataset, a sanity check should follow: by identifying the words most associated with the positive and negative class and manually reviewing these words and how they are used in the dataset, users of sentiment analysis tools can rule out common sources of error. For example, if slurs commonly appear in sentences classified as negative, then such a closer analysis might reveal that they are used in a non-derogatory, reclaimed way.
3. **Mitigation:** Any such issues identified in this step need to be mitigated before the results can be trusted. In the case of lexicon-based methods, the lexicon can be directly edited, while in the case of deep learning models, more complex and resource intensive methods may be necessary, such as CAD (Meade et al., 2021); a more practical solution for the user may be to try a different model and see if the issue persists. This also means that when choosing a tool, researchers may need to trade off flexibility and interpretability, the strengths of lexicon-based tools, against accuracy and sophistication, the strengths of deep learning-based models. When analysing data from marginalised communities, it seems likely the transparency of the lexicon-based approaches makes them the most suitable choice.

## Conclusion

In this paper, we have illustrated some of the potential pitfalls facing researchers using automated sentiment analysis tools. We found that both lexicon and ML-based approaches to sentiment analysis can result in inaccurate results when comparing queer and non-queer identities, and in some cases, this amounted to a systematic bias against, for example, queer women or transgender people. We indicate how these findings likely extend to other marginalised identities beyond the LGBTQ community. We explored how ML-based approaches promise more sophisticated analyses, but this comes at the expense of making the source of bias harder to identify and harder to mitigate; and how on the reverse, lexicon-based approaches are easy to debias but offer less sophisticated language processing. We caution that the issues associated with automated sentiment analysis will be particularly disruptive when analysing text from and about individuals belonging to different identity groups, as use of certain terms by one group and not the other may result in spurious differences. Use of automated tools may speed up aspects of research, but without careful research into the appropriate choice of tool this may prove to be a false economy, if steps are not taken to mitigate the impact of predictive bias.

## Notes

1 Understood as a bias against LGBTQ + individuals

2
https://www.ibm.com/uk-en/cloud/watson-natural-language-understanding

3
https://www.elsevier.com/en-gb/solutions/scopus

4
https://proquest.libguides.com/assia/content

5 Although not all non-binary people identify as transgender (Rimes et al., 2019).

6 We use ‘they’ pronouns for all two-spirit examples but there are those who identify as two-spirit men and two-spirit women and may prefer other pronouns; traditions vary across tribes.

7
https://www.lexalytics.com/semantria

## Pronoun Use

The data set can also be used to measure the impact of non-binary pronoun choice: both ‘themself’ and ‘themselves’ are reflexive pronouns commonly used by the non-binary community, and we include sentences with both (i.e. all non-binary variations were included once with ‘themself’ and once with ‘themselves’).

## Additional Information on Included Terms

The terms list includes additional plurisexual identities (those attracted to multiple genders), namely ‘pansexual, fluid’ in addition to ‘bisexual’ from Dixon et al. (2018). Plurisexual individuals often face discrimination from both queer and non-queer people, known as double discrimination (Mereish et al., 2017). The data set therefore allows for the detection of bias against plurisexual individuals (as well as against the individual sexualities). Reiterating a point we make above, when no sexuality is given, the norm (monosexuality) will be assumed. Note that whilst queer is widely used by plurisexual people, it is also used by monosexual people (Kolker et al., 2020), so we do not consider it plurisexual. We also include in the sexuality list two ace-spectrum identities (Hille et al., 2020), namely ‘asexual, demisexual’, which will enable exploration of ace-phobic bias.

In order that the data set be useful for detecting bias against plurisexual identities, we combined terms relating to sexuality presentation style (e.g. ‘butch’), where monosexuality is assumed, with the term ‘bisexual’. Our research at the time suggested ‘stud’ was used by black women who exclusively love women (Lane-Steele, 2011), and as 'bisexual' is a non-ethnicity specific term whilst 'stud' is ethnicity specific, we felt it would be inappropriate to combine the terms. However, there has been a movement on social media bringing attention to bisexual studs. We welcome researchers to use our code to generate sentences including 'bisexual stud', and further welcome inclusion of more ethnicity specific terms in our data set.

When constructing the data set and in our analyses, we treat ‘queer, LGBT’ and ‘LGBTQ’ as sexuality terms. We felt this was the most appropriate way to include these umbrella terms without introducing a fourth identity term category (and thus effectively tripling the size of the data set). Although they are intended to encompass all queer identities, it is our intuition that the cisgender norm is often assumed despite the inclusion of T for transgender in the two acronyms. Indeed, LGBT(Q) is often used in direct opposition to straight, despite transgender straight people being part of the LGBT community. Another researcher might prefer to exclude variations with LGBT and LGBTQ when considering non-transgender identities (we treat these as ‘assumed cisgender’ identities).
